# The Common Accidents of Every-Day Life

**Published:** 1887-11-26

**Authors:** Robert A. P. Forrester


### The Common Accidents of Every-day Life.

By Robert A. P. Forrester, B.A.Dub., M.B. and C.M.Edin.

### Burns and Scalds.

*Childhood.*—Numerous instances of injury and death from the above might be cited, for of all accidents these are the most commonly fatal in civil life. The *causes* of burns and scalds exercise a certain influence on the progress of the case, and should always be taken into account in giving an opinion as to the termination of the same. The most common and fatal is the prolonged contact of fire to the body by the burning of the clothes. Gas explosions, though not causing deep burns, should not on that account be lightly thought of, for severe consequences often ensue. The contact of molten metal causes very deep burns, which almost always slough.

Scalds are usually not so severe as burns, because the fluid does not remain for so long a time in contact with the body; but the extent of surface implicated may be greater and render the accident as grave.

Children are particularly liable to be the victims, and there are few wards in our hospitals without their youthful contingent of "burns and scalds." These injuries are generally accompanied with great pain and suffering, and when large surfaces of the body are involved, great deformity may follow in spite of the care and attention devoted to the treatment. Nothing so much tests a nurse's patience and devotion as the proper management of a bad burn case. A few words, therefore, about the skin might not be out of place. The skin is one of the excretory organs of the body, and is concerned with the elimination of a small quantity of salts, a little carbonic acid, and a large quantity of water in the form of perspiration. It consists of two layers: an upper one without blood-vessels, called the epidermis or cuticle, and a lower, vascular (*i.e.*, with blood vessels), a very sensitive one, called the *cutis vera*, or true skin. The former, also called scarf skin, is the outer covering, and protects the true skin. Its thickness varies in different localities from 1-10th to 1-200th inch; it is thickest where it is most frequently exposed to pressure, *e.g.*, the sole of the foot and the hands of working men. When the cuticle is removed the true skin comes into view; it is a much more complicated structure than the epidermis, being highly vascular, with numerous nerves, sweat, and sebaceous glands distributed through it.

It has been estimated that while seven grains of matter left the body by the lungs, as much as eleven grains escaped through the skin per minute. This amount though varies extremely. Of the whole amount part passes off at once as watery vapour containing volatile matters, while part remains on the skin as a fluid—the former is known as *insensible* perspiration, the latter as *sensible*. The proportion, however, of the insensible to the sensible perspiration depends on many causes, *e.g.*, the rapidily of the secretion in reference to the dryness, temperature, and amount of movement of the surrounding atmosphere. When the air is hot and dry, the greater is the amount of the *sensible* perspiration which is converted by evaporation into the *insensible* condition, but when the air is cool and moist a large amount of the perspiration may remain on the skin as sweat.

When the function of the skin is largely interfered with, as in an extensive burn, it is natural to expect that those other organs of the body connected with excretion will be involved also to their detriment, perhaps on account of the extra strain put upon their capabilities.

In burns and scalds we hear then of other complications, *e.g.*, pleurisy, inflammation of the lungs, ulceration of the duodenum, and other parts of the intestine. Death may occur from any of these causes, and not be due immediately to the primary injury, or from shock and collapse soon after the accident. Dupuytren, a French surgeon, divided burns into six degress, according to their depth,

The first degree is characterised by a superficial redness, with often a good deal of pain attending it. Such an accident as a gas explosion may cause it, and as the contact of the flame is only momentary, no depth of surface is implicated. Being extensive, perhaps more is to be feared from the depressing influence on the heart and nervous system than rom the injury itself.

In the second degree we find the formation of bullæ or vesicles. The injury here is deeper, and the sudden determination of blood to the part has been relieved by the transudation of serum through the walls of the blood vessels. The cuticle in this instance has been separated from the cutis, and no longer affords it protection. The true skin is partially destroyed in the *third* degree. In the *fourth* degree the whole of the true skin is destroyed, and the tissues underneath involved. It is not always an easy matter to distinguish at the time these two degrees of burning, but those who have much experience in these cases can generally do so. When the true skin is all destroyed it is difficult to prevent contraction during the process of healing. In the *fifth* degree more or less of the parts under the skin are destroyed; and in the *sixth* degree, fortunately very rare, we have a total charring.

When consulted as to the prospects of a burn case, one must take into consideration the age of the patient, the extent of the burn, and its depth. Children are very liable to the complications above-mentioned, and very old people are ill-calculated to withstand the effects of such an injury. The extent of a burn is an important point. When a large surface is involved, even when only for the first or second degree, the shock to the system and the great pain make recovery doubtful, and where the true skin is deeply burnt, and great subsequent suppuration occurs, there is little hope of recovery. When the chest is much injured the chances are less, especially in children.

*Symptoms.*—Of these the most important is *pain*. The pain of burning is the most terrible of all, and it is fortunate that life cannot long continue under the pressure of such torture. I remember a little boy aged three years being admitted to the hospital where I was house surgeon. He was literally burnt from head to foot, and in some parts, especially over the chest and thighs, very severely. His screams and shrieks were heartrending. I was compelled during the dressing of his wounds to keep him under the influence of chloroform, and had to continue its administration ever and again until his death from shock, about two hours after admission. The pain necessarily, however, varies, but in nearly every case is severe, and in adults ought to be met with a dose of opium. The constitutional symptoms are extreme prostration—especially when the pain has been very severe—pallor, and terror, which is very apparent in young children. Delirium is also very frequent. Rigors also occur. The pulse is quick and weak, tongue dry, and thirst great. The patient often dies at this stage, death being preceded by convulsions or delirium. This prostration may last for a day or two, and is followed by a period of reaction, when symptoms of inflammation may be looked for. If you find vomiting, with diarrhœa and blood in the motions, ulceration of the duodenum has probably occurred. Inflammation of the lungs may be expected if we find cough and breathlessness.

The sloughs begin to separate now, and suppuration commences. This tends to weaken the patient, and the most prominent symptom to meet is the extreme weakness. Meanwhile, the wound has gone on in the healing process, and may terminate favourably, or the cicatrisation may be arrested and an ulcer remain, which often is extremely difficult to cure. (*To be continued*).

